# Enhancer-driven alternative promoters of imprinted genes

**DOI:** 10.1371/journal.pone.0208421

**Published:** 2018-11-30

**Authors:** Joomyeong Kim, Bambarendage P. U. Perera, Subash Ghimire

**Affiliations:** Department of Biological Sciences, Louisiana State University, Baton Rouge, United States of America; Texas A&M University, UNITED STATES

## Abstract

In the current study, we characterized the expression and histone modification profiles of the alternative promoters found within imprinted *Igf2r*, *Mest*, *Zac1*, *Peg3*, *Snrpn* and non-imprinted *Myc* loci. In terms of expression pattern, the alternative promoters are highly tissue-specific, which is in a stark contrast to the ubiquitous expression of the corresponding main promoters. The alternative promoters are associated with the histone modification mark H3K4me1, but not with H3K4me3, which is frequently associated with the main promoters. Phylogenetic analyses also indicated that the majority of the alternative promoters are unique to the mammalian lineage, further suggesting the recent formation of these promoters during mammalian evolution. Overall, this study suggests that the alternative promoters of imprinted loci may have been derived from enhancers in recent evolutionary times and co-evolved with the genomic imprinting mechanism.

## Introduction

In mammalian genomes, a subset of genes are expressed mainly from one allele due to an epigenetic mechanism termed genomic imprinting, by which one allele is repressed by DNA methylation and histone modifications [1, 2]. About 100 to 200 genes are known to be imprinted in mammalian genomes, and these genes are usually expressed in early-stage embryo, placenta and brain [1–3]. Thus, it has been predicted that imprinted genes may play significant roles in fetal development and animal behaviors. Consistent with this, mutations in these imprinted genes have very similar functional outcomes, such as changes in fetal growth rates and also in animal nurturing behaviors [1, 2]. Interestingly, genomic imprinting is found only within the eutherian lineage that has an unusual reproduction strategy involving viviparity and placentation. Thus, it has been hypothesized that genomic imprinting may have co-evolved with the reproduction scheme of placental mammals to control the dosage of the genes that play important roles in the reproduction-related physiology and behaviors [4–7].

Imprinted genes also tend to be clustered in specific regions of chromosomes, forming imprinted domains. The imprinting of several genes in a given domain is controlled through small genomic regions, termed Imprinting Control Regions (ICRs) [1, 2]. ICRs obtain allele-specific DNA methylation during gametogenesis, which is then maintained throughout the lifetime after fertilization [1, 2]. One of key questions regarding genomic imprinting has been how this allele-specific DNA methylation is established during gametogenesis. According to the results from several imprinted domains, alternative promoters for very low abundant transcripts may play key roles in this process [8–13]. These alternative promoters are usually functional during oogenesis, and trigger transcription. Also, these alternative promoters are all located upstream of their ICRs, thus the transcription usually traverses through the ICRs during oogenesis. This passing of RNA Pol II has been shown to be important for setting up oocyte-specific DNA methylation on the ICRs. Either deletion of the alternative promoters or truncation of the alternative transcripts usually causes defects in setting up oocyte-specific DNA methylation on the ICRs [8–13]. Recent studies further suggest that the recruitment of the *de novo* DNA methyltransferase DNMT3A to ICRs may require one particular histone modification, H3K36me3, which is formed as part of the transcription elongation process by RNA Pol II [14].

In the current study, we sought to identify additional alternative promoters from imprinted loci by performing a series of Next Generation Sequencing (NGS)-based 5' Rapid Amplification of cDNA Ends (RACE) experiments. With this series of experiments, we have identified several alternative promoters, and subsequently analyzed their expression and histone modification profiles. According to the results, these alternative promoters share several features with transcriptional enhancers, and are likely formed during recent mammalian evolution. Thus, we predict that these alternative promoters might have been derived from enhancers and subsequently co-evolved with the genomic imprinting mechanism.

## Results

### Alternative promoters identified from 5’RACE experiments

**Fig 1:**
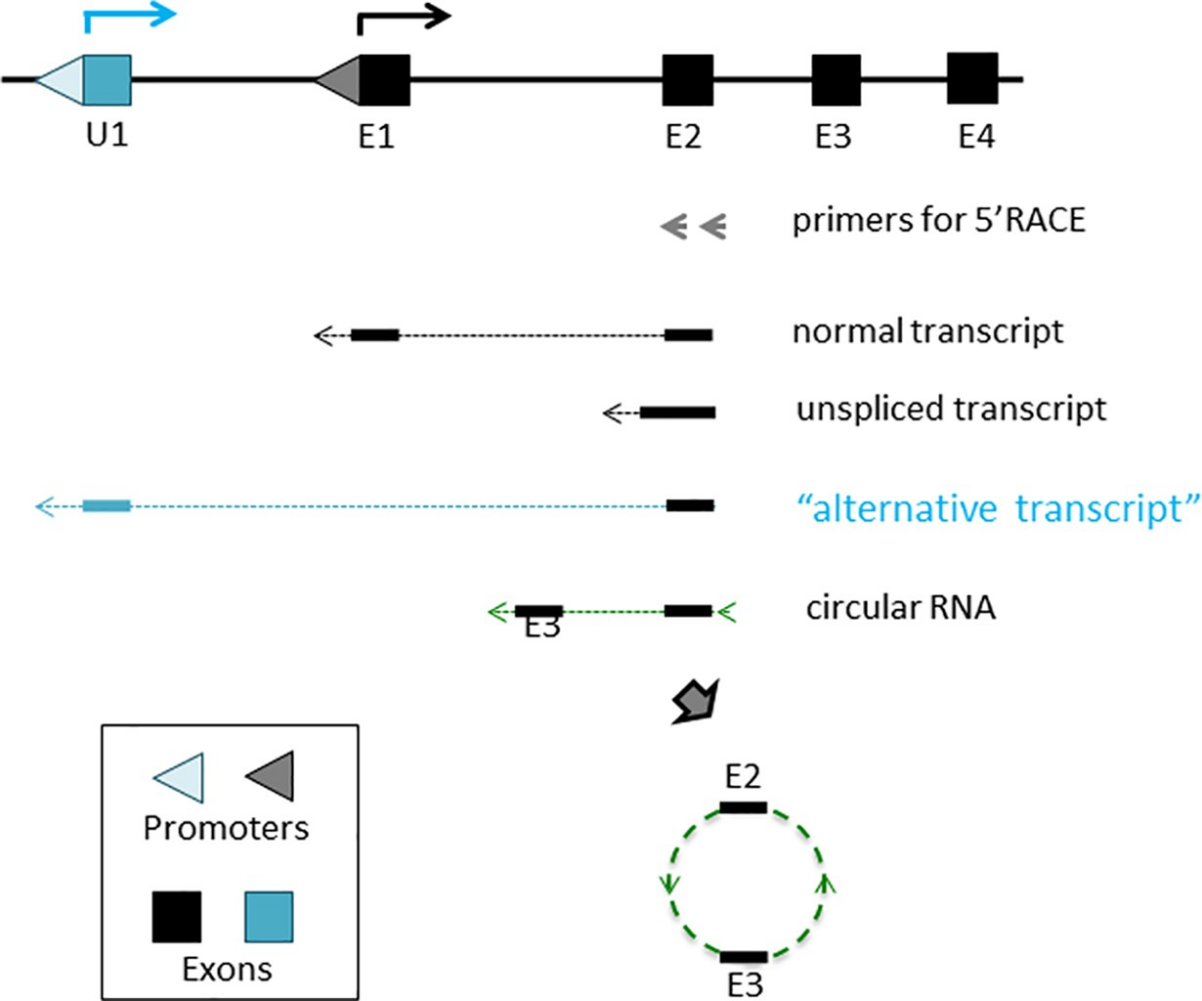
Alternative promoters/exons identified through 5'RACE experiments. Schematic representation of 5'RACE experiment. Total RNA isolated from tissues was first reverse-transcribed with the gene-specific primers that are derived from the 2nd exons of individual genes (grey arrows). These cDNAs were further modified with G-tailing (arrowheads). The subsequent cDNAs were amplified for NGS runs. Examination of the sequence reads identified several different types of transcripts: normal transcripts with a proper joining of two known exons (E1, E2), unspliced transcripts, circular RNA transcripts, and alternative transcripts derived from upstream alternative promoters/exons (U1).

We performed a series of NGS-based 5’ RACE experiments to identify unknown alternative promoters that are localized upstream of the known promoters of imprinted genes (Fig 1). For this series of experiments, we isolated total RNA from the hypothalamus, neonatal heads, ovary, and testis of the mouse. The isolated total RNA was subsequently reverse-transcribed with the gene-specific primers that were derived from the 2nd exon of imprinted genes, including *Snrpn*, *Zac1*, *Gtl2*, *Dlk1*, *Igf2r* and non-imprinted *Myc*, which served as a control. These cDNAs were further modified with G-tailing, which were then amplified for NGS runs. We obtained total 2.5 million reads from this series of experiments. Inspection of these raw reads derived the following immediate conclusions. First, the majority of the sequence reads still belonged to the two categories of the transcripts: normal transcripts with a proper joining of two known exons, Exon1 (E1) and Exon2 (E2), and unspliced transcripts. For the majority of the six tested genes, these two categories of transcripts made up more than 90% of the mapped sequence reads from each of the four sequenced libraries (S1 File). Second, several circular RNA transcripts have been also identified from the following genes, *Zac1*, *Dlk1*, and *Igf2r*. In the case of these genes, the sequences of Exon2 were joined to the sequences of either the 3'-side of Exon2 itself or the downstream exons. These circular RNAs have been recently reported in a separate study [15]. Third, alternative transcripts starting from upstream alternative promoters/exons (U1) have been identified from the following four genes: *Snrpn*, *Zac1*, *Igf2r*, and *Myc* (S1 and S2 Files). According to the results, the sequence reads corresponding to the alternative transcripts of *Snrpn* and *Zac1* were mapped to the very same genomic regions that had been previously characterized as alternative promoters or exons by others [9–11], thus confirming the feasibility of the NGS-based 5’RACE approach used for the current study. On the other hand, the sequence reads corresponding to the alternative transcripts of *Igf2r* and *Myc* were mapped to the genomic regions with no prior indications for potential exons, thus suggesting that the identified regions may represent previously unknown alternative promoters/exons for *Igf2r* and *Myc*. In terms of abundance, the alternative transcript (U1) of *Igf2r* accounted for 7.2% of the transcripts from the ovary (727 out of 9,974 total reads), while the alternative transcript (U1) of *Myc* made up 2.2% of the transcripts from the neonatal heads (4,428 out of total 201,504 reads) (S1 File). Thus, both newly identified transcripts are considered to be minor transcripts for the corresponding two genes. Overall, the NGS-based 5'RACE approach has successfully identified several alternative promoters for the tested genes, including the previously unknown ones for the *Igf2r* and *Myc* loci.

### Alternative promoters from the mouse *Igf2r* and *Myc* locus

**Fig 2:**
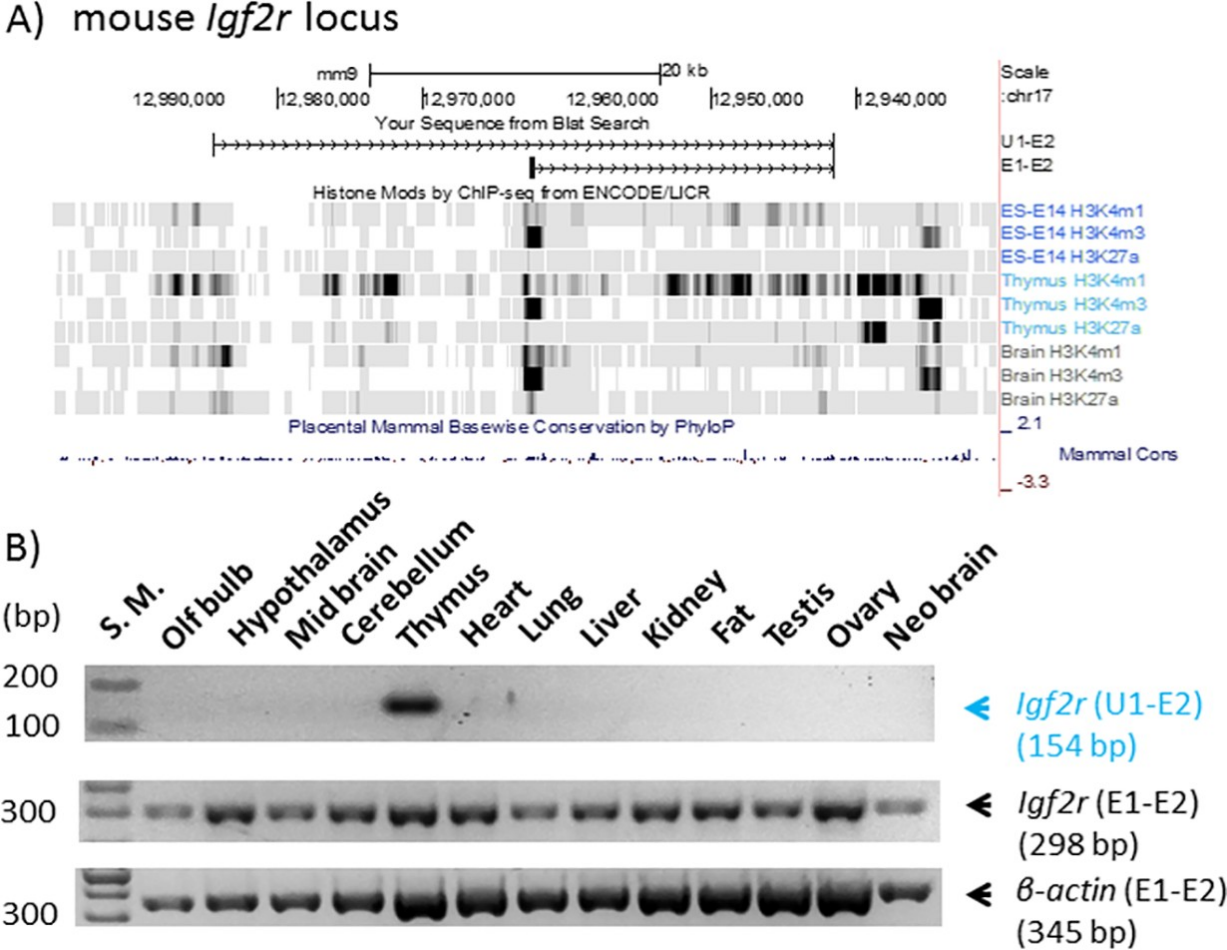
Alternative promoter/exon identified from the mouse *Igf2r* locus. (**A**) Genomic structure and histone modification profiles of an alternative promoter (U1) and the main promoter (E1) of mouse *Igf2r*. Genomic positions of U1, E1 and E2 (exon 2) are indicated with vertical lines on the genome browser of UCSC. Histone modification profiles (H3K4me1, H3K4me3, H3K27ac) from embryonic cells (ES), thymus, and brain are presented along with sequence conservation profiles (Mammal Cons). (**B**) Expression profiles of U1 and E1 of *Igf2r*. A set of cDNAs derived from 13 individual tissues was used for RT-PCR surveys monitoring the expression patterns of U1 and E1 promoters. Expression profile of *β*-Actin was included as a control.

The newly identified alternative promoters of *Igf2r* and *Myc* were further characterized in terms of their expression and histone modification profiles. The expression patterns of the identified alternative promoters were first determined through performing RT-PCR-based surveys (Fig 2). For this series of surveys, we isolated total RNA from the 13 individual tissues of the mouse, which were then converted into cDNA for RT-PCR surveys. For each locus, we designed two sets of primers: the first one for the alternative promoter targeting the transcripts starting from U1 through E2 (U1-E2), and the second one for the main promoter amplifying the transcripts starting from E1 through E2 (E1-E2). In the case of *Igf2r*, the alternative transcript starting from the U1 promoter was detected mainly from the thymus, while the main transcript starting from the E1 promoter was detected from all the tissues tested so far (Fig 2B). Thus, the expression patterns of the alternative promoter are quite tissue-specific, which is in a stark contrast to the observed ubiquitous expression patterns of the main promoter. This series of RT-PCR surveys were repeated three times for each of two biological replicates, which derived similar outcomes. The U1 alternative promoter of *Igf2r* is located 22-kb upstream of its corresponding E1 main promoter. The genomic region surrounding this alternative promoter was inspected with the histone modification profiles, which are publically available as the dataset of the ENCODE project [16]. According to the results, the genomic region surrounding this alternative promoter is marked mainly with two histone marks, H3K4me1 and H3K27ac, which are known to be associated with active enhancers (Fig 2A) [16, 17]. On the other hand, a typical promoter is usually associated with another histone mark, H3K4Me3, which is shown in the main promoter of *Igf2r*. However, this modification is not detected at all from the genomic region surrounding the identified U1 alternative promoter. Thus, the U1 alternative promoter of *Igf2r* may be regarded as an enhancer rather than a promoter according to the associated histone modification profiles.

**Fig 3:**
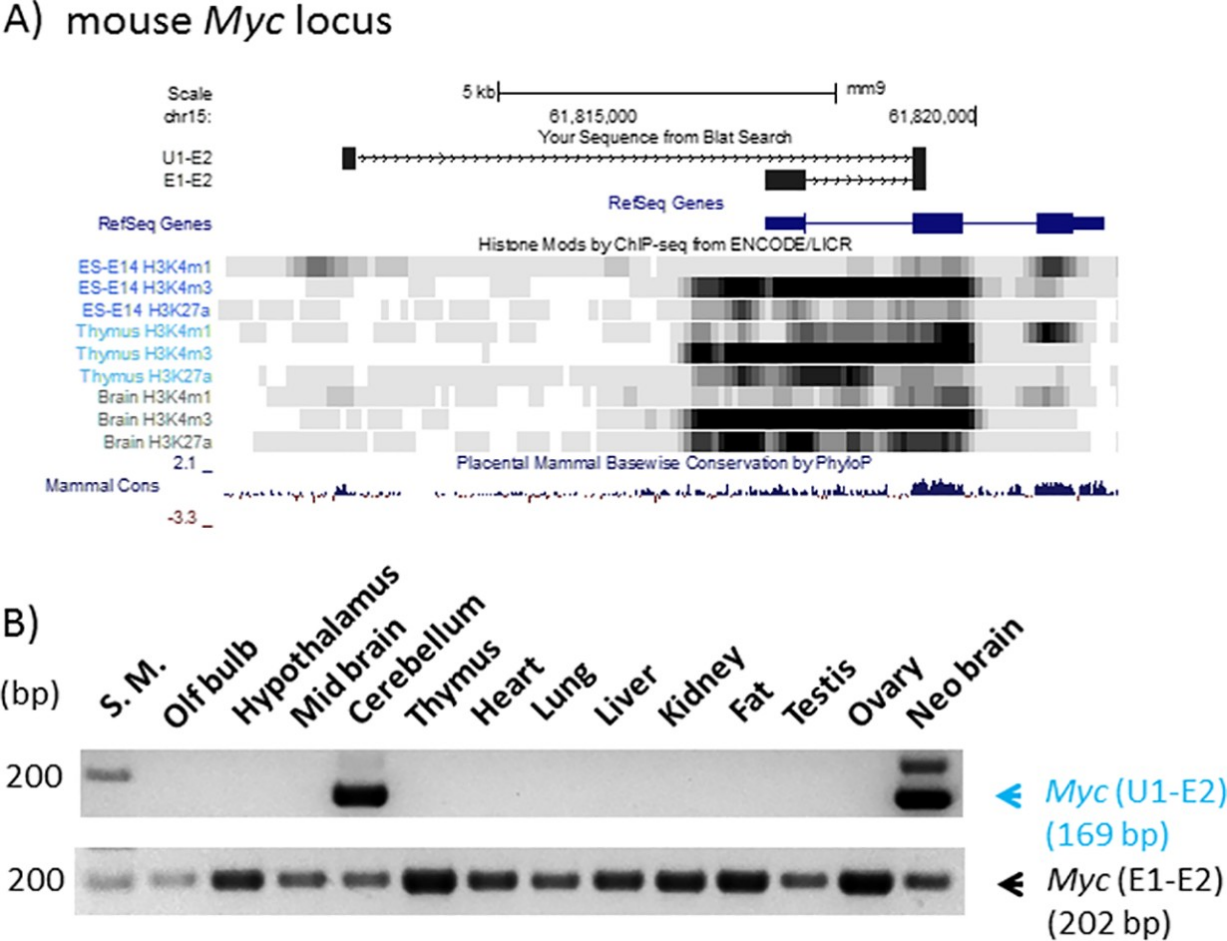
Alternative promoter/exon identified from the mouse *Myc* locus. (**A**) Genomic structure and histone modification profiles of an alternative promoter (U1) and the main promoter (E1) of mouse *Myc*. Genomic positions of U1, E1 and E2 (exon 2) are indicated with black boxes on the genome browser of UCSC. Histone modification profiles (H3K4me1, H3K4me3, H3K27ac) from embryonic cells (ES), thymus, and brain are presented along with sequence conservation profiles (Mammal Cons). (**B**) Expression profiles of U1 and E1 of *Myc*. A set of cDNAs derived from 13 individual tissues was used for RT-PCR surveys monitoring the expression patterns of U1 and E1 promoters.

For the proto-oncogene *Myc* locus, we also performed a similar series of analyses (Fig 3). According to the results, the expression of the alternative transcript was detected in adult cerebellum and neonatal brain, displaying neuron-specific expression patterns. In contrast, the expression of the main transcript was detected throughout all the tissues tested (Fig 3B). Thus, as shown in *Igf2r*, the alternative promoter of *Myc* also exhibited a very tissue-specific expression pattern, while the main promoter showed a ubiquitous expression pattern. Similar to the *Igf2r* locus, the alternative promoter of *Myc* is localized 8-kb upstream of the main promoter, and also associated with the H3K4me1 mark, but not with the H3K4me3 mark. Thus, the alternative promoter of *Myc* may also be considered an enhancer rather than a promoter. In fact, the 200-kb upstream of the *Myc* locus has been recognized as a 'super enhancer' region housing a large number of evolutionarily well conserved putative enhancers, which are associated with the H3K4me1 and H3K27ac marks [18, 19]. The identified U1 alternative promoter is likely one of these enhancers, which is further supported by its high levels of sequence conservation among all the mammals, as shown in the mammalian conservation plot (Fig 3A). Overall, this series of analyses revealed that the two identified alternative promoters from *Igf2r* and *Myc* are highly tissue-specific, which are quite different from the ubiquitous expression patterns observed from their corresponding main promoters. The alternative promoters are also associated with the histone mark H3K4me1, which is known to be associated with enhancers.

### Tissue-specific expression and histone modification profiles of alternative promoters

**Fig 4:**
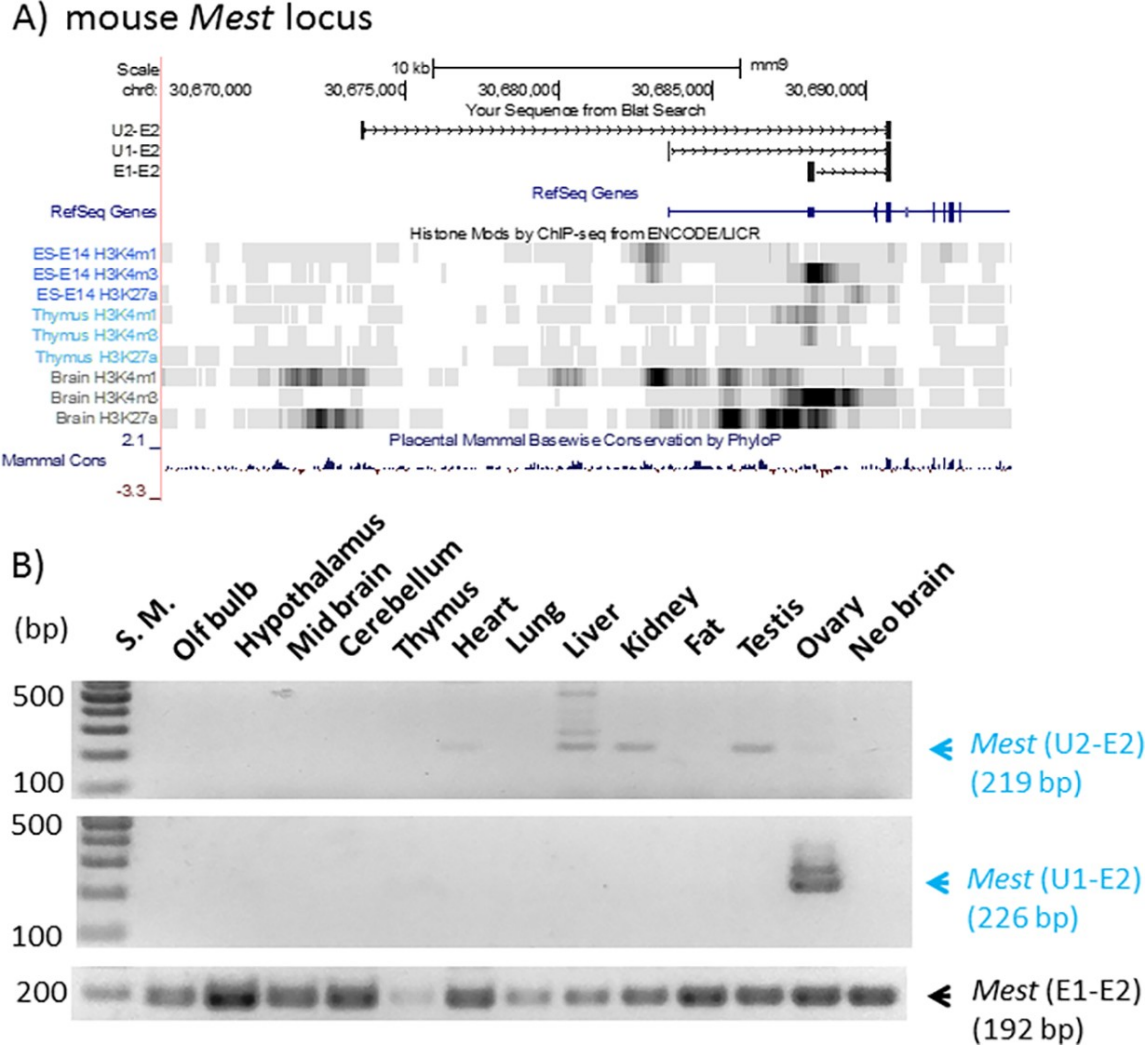
Alternative promoters/exons of the mouse *Mest* locus. (**A**) Genomic structure and histone modification profiles of two alternative promoters (U1 and U2) and the main promoter (E1) of mouse *Mest*. Genomic positions of U1, U2, E1 and E2 (exon 2) are indicated with vertical lines on the genome browser of UCSC. Histone modification profiles (H3K4me1, H3K4me3, H3K27ac) from embryonic cells (ES), thymus, and brain are presented along with sequence conservation profiles (Mammal Cons). (**B**) Expression profiles of U1, U2, and E1 of *Mest*. A set of cDNAs derived from 13 individual tissues was used for RT-PCR surveys monitoring the expression patterns of U1, and U2, and E1 promoters.

We extended the analyses described above to the previously identified alternative promoters of the imprinted genes, including *Mest*, *Zac1*, *Peg3*, and *Snrpn* (Fig 4 and S3, S4, S5 Files). First, the mouse *Mest* locus is known to contain two alternative promoters, U2 and U1, that are localized 14 and 5-kb upstream of the main promoter, respectively (Fig 4). As seen in *Igf2r* and *Myc*, these two alternative promoters are also associated with H3K4me1 and H3K27ac, but not with H3K4me3. On the other hand, the main promoter of *Mest* is closely associated with H3K4me3 and H3K27ac (Fig 4A**)**. In terms of expression patterns, the alternative transcript starting from the U2 promoter was detected at very low levels from the heart, liver, kidney, and testis, whereas the alternative transcript from the U1 promoter was detected only from the ovary (Fig 4B). On the other hand, the main transcript was detected ubiquitously from all the tissues, although the expression levels in the thymus was relatively low.

**Fig 5:**
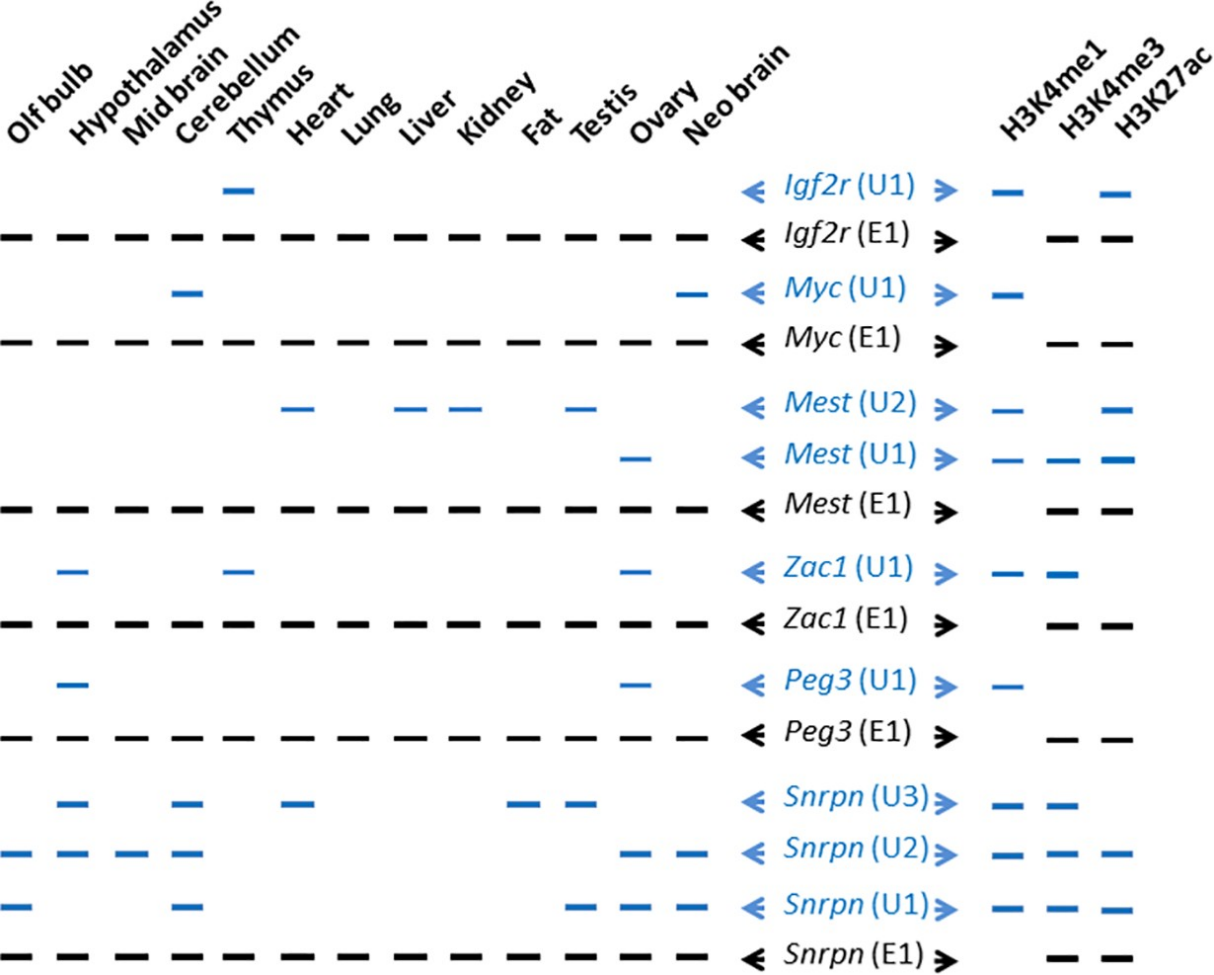
Expression patterns of alternative promoters of imprinted genes. This schematic diagram on left summarizes the results derived from RT-PCR-based expression analyses of imprinted genes using a set of cDNA prepared from 13 individual tissues. The schematic diagram on right summarizes the histone modification profiles obtained from a previous study [17]. For each imprinted gene, the main promoter-driven expression is indicated with a black line in a given tissue, whereas the alternative promoter-driven expression is indicated with a blue line. The names of alternative and main promoters for each gene are indicated on middle. The actual RT-PCR results used for this schematic summary are presented in Figs 2, 3 and 4 and also in Supporting information (S3, S4, S5 Files).

Second, we repeated this series of analyses on the three remaining genes, *Zac1*, *Peg3*, and *Snrpn*, and the actual results are available as Supporting information (S3, S4, S5 Files). Along with the other loci described above, the patterns observed from these three loci can be summarized as follows (Fig 5). In terms of expression patterns, the alternative promoters tend to be very tissue-specific, whereas the main promoters are ubiquitous. In particular, the two tissues, the hypothalamus and ovary, are the most frequent tissues where the alternative promoters of imprinted genes are active and thus trigger alternative transcription. It is worthwhile to mention that the alternative promoters for *Zac1*, *Peg3*, and *Snrpn* are highly active in growing oocytes, and that the transcription from these alternative promoters are primarily responsible for establishing the oocyte-specific DNA methylation on the downstream main promoters [8–13]. Thus, the detection of these alternative transcripts in the ovary is consistent with the previous observations. It is interesting to point out the fact that these alternative promoters are also active in the hypothalamus, although the biological reasons for their shared expression in the hypothalamus are currently unknown. In terms of histone modification profiles, the alternative promoters tend to be closely associated with the H3K4me1 mark, which is quite different from the close association of the H3K4me3 mark with the main promoters. In the case of *Zac1* and *Snrpn*, the alternative promoters are also associated with both H3K4me1 and H3K4me3 marks. Overall, this series of the extended analyses again confirmed the observed patterns: the alternative promoters tend to be very tissue-specific and associated with the histone modification mark H3K4me1 (Fig 5).

### Recent formation of alternative promoters

**Fig 6:**
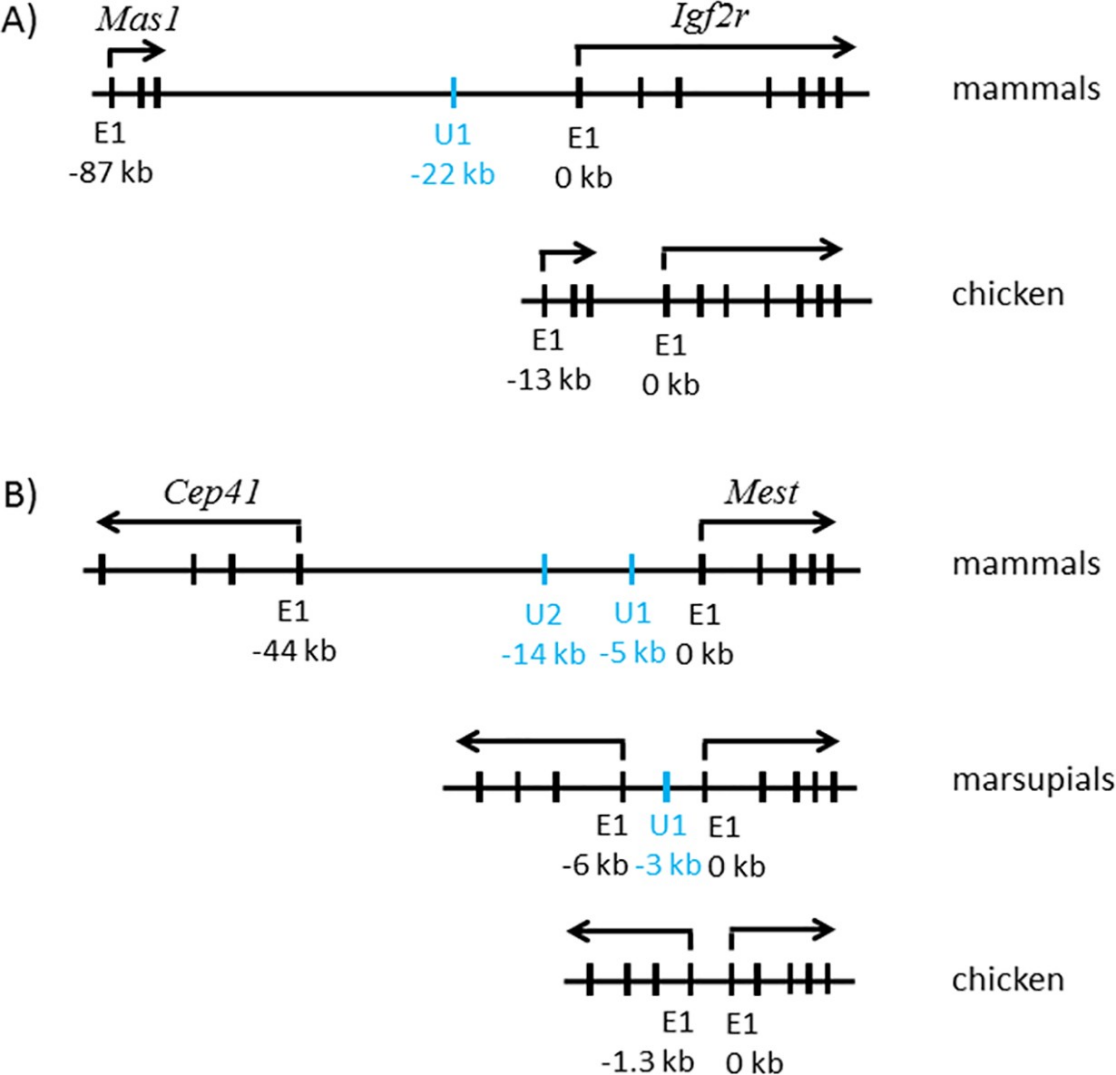
Comparison of the genomic loci harboring *Igf2r* and *Mest* among mammals, marsupials, and chicken. (**A**) The 175-kb genomic locus surrounding mouse *Igf2r* is shown as a representative locus for mammals (chr17:12,874,015–13,049,382, mm9), whereas the 69-kb homologous locus containing chicken *Igf2r* is shown as a representative for non-mammalian vertebrates (chr3:44,771,696–44,840,491, galGal4). The relative genomic positions of the promoter of *Mas1* (proto-oncogene Mas1) and the U1 alternative promoter of *Igf2r* are indicated below with the E1 main promoter of *Igf2r* as a reference position, 0 kb. (**B**) The 96-kb genomic region surrounding mouse *Mest* is shown as a representative for mammals (chr6:30,602,691–30,698,764, mm9); the 58-kb genomic region of opossum *Mest* as a representative for marsupials (chr8:190,407,190–190,465,490, monDom5); and the 13-kb genomic region of chicken *Mest* as a representative for the non-mammalian vertebrates (chr1:921,494–934,578, galGal4). The genomic positions of the promoter of *Cep41* (Centrosomal protein of 41 kDa) and the two alternative promoters (U1 and U2) of *Mest* are shown below relative to that of the E1 main promoter of *Mest*.

The evolutionary origin of the identified alternative promoters was also characterized with comparative genomic approaches (Fig 6). Among the five imprinted loci with the alternative promoters, the two loci, *Zac1* and *Peg3*, are unique to the eutherian lineage, since the orthologous loci of these two imprinted genes cannot be traced in marsupials and monotremes and also in the other vertebrates. In contrast, the other three loci, *Igf2r*, *Mest*, and *Snrpn*, are traceable and well conserved among all the vertebrates. First, the 175-kb genomic interval containing *Igf2r* and *Mas1* (proto-oncogene Mas1) is well conserved among all the eutherian mammals in terms of gene orientation and distance (Fig 6A). A similar syntenic interval is also found even in the avian, including chicken. Detailed inspection of the genomic intervals, however, provided several differences between individual vertebrates. In particular, the size of this genomic interval in chicken, 69 kb in length, is much shorter than that of mammals and marsupials, on average 180 kb in length. In particular, the intergenic region between *Igf2r* and *Mas1* becomes much shorter in chicken, only 13 kb in length, compared to that in mammals, 87 kb in length. The U1 alternative promoter localized 22-kb upstream of the main promoter cannot be found in chicken, although the homologous sequence could be located in the marsupial and platypus genomes. Thus, it is likely that the U1 alternative promoter may have been formed in recent evolutionary times after the split of the mammalian and avian lineages.

Second, the 96-kb genomic interval containing *Cep41* (Centrosomal protein of 41 kDa) and *Mest* is also well conserved among all the mammals, including marsupials and platypus, and also even in the avian lineage (Fig 6B). Similar to the *Mas1*-*Igf2r* genomic interval, the *Cep41*-*Mest* interval also showed quite dramatic size differences between individual vertebrates: 96 kb in mammals, 59 kb in marsupials, and only 13 kb in chicken. The homologous sequence of U1 can be found in the platypus, but not in the avian genomes, thus suggesting the recent origin of this alternative promoter in the mammalian lineage. Furthermore, the homologous sequences of U2 cannot be found even within some of mammals, such as marsupials and platypus, thus indicating the more recent formation of this promoter than the U1 promoter. A similar situation appears to have occurred in the case of the *Snrpn* locus. The three alternative promoters (U1 through U3) of *Snrpn* are believed to have been formed through genomic duplication only in the muroid genomes, including mouse, rat and Chinese hamster. This is based on the fact that the other mammals have only one alternative promoter U1, which is localized about 100-kb upstream of the main promoter (S6 File). Overall, the phylogenetic analyses described above indicated that the alternative promoters of imprinted genes are unique to the mammalian lineage, although the main promoters could be found beyond the mammalian lineage. Thus, the majority of the identified alternative promoters are predicted to have been formed in recent evolutionary times.

## Discussion

In the current study, we characterized the expression and histone modification profiles of several alternative promoters that are localized upstream of the main promoters of imprinted genes. According to the results, the alternative promoters are very tissue-specific, and associated with the histone modification mark H3K4me1. Also, the results from phylogenetic analyses indicated that the majority of the alternative promoters are unique to the mammalian lineage, thus suggesting the recent formation of these promoters during mammalian evolution.

The newly identified alternative promoters of *Igf2r* and *Myc* along with those of *Mest*, *Zac1*, *Peg3*, *Snrpn* share a set of unique features that are distinct from those observed from the corresponding main promoters. First, the expression patterns of the alternative promoters are quite tissue-specific, including thymus-specific expression of *Igf2r*, neuron-specific expression of *Myc*, hypothalamus and ovary-specific expression of *Peg3* and *Zac1* (Fig 2, Fig 3, Fig 5). Second, the majority of these alternative promoters are associated with H3K4me1, which is again quite different from the typical association of mammalian promoters with H3K4me3. In fact, the histone modification mark H3K4me1 is a hallmark for transcriptional enhancer [16, 17]. Finally, the relative genomic positions of these alternative promoters are also quite distant from the main promoters and coding exons. Given these shared features, it is likely that the identified alternative promoters might have been derived from enhancers or may be actual enhancers with occasional promoter function. This scenario is most likely given the recent observation that many enhancers recruit RNA Pol II machinery [20, 21]. Yet, the recruited RNA Pol II tends to transcribe short-length nascent transcripts from some of active enhancers [20, 21]. Therefore, it is reasonable to predict that some of the enhancers within imprinted loci may have become alternative promoters or play dual functions with occasional promoter activity.

According to the results from phylogenetic analyses, the majority of the alternative promoters are unique to the mammalian lineage (Fig 6). In the case of *Igf2r*, the U1 alternative promoter is not found in the other mammals and vertebrates. This is also the case for the *Mest* locus: the U1 and U2 alternative promoters are unique to the eutherian mammals. In contrast, the main promoters of both loci are conserved in the other vertebrates. This suggests that some of the imprinted loci may predate the speciation of the mammalian lineage and also the advent of the genomic imprinting mechanism. Given their recent origin in the mammalian genomes, it is also reasonable to predict that the advent of the alternative promoters may have coincided with that of the genomic imprinting mechanism. This is further supported by the fact that the alternative promoters of *Zac*1, *Peg3*, and *Snrpn* play critical roles in establishing the allele-specific DNA methylation on the corresponding main promoters during oogenesis [8–13]. However, given the small number of genes tested in the current study, this prediction is very speculative at the moment and requires thorough investigation in the future. On the other hand, the ovary-specific expression of the U1 promoter of *Mest* appears to be also consistent with the pattern observed from the other paternally expressed genes, displaying the expression of their alternative promoters in oocytes (Fig 5). Thus, it should be interesting to test whether the U1 promoter of *Mest* also plays a similar role in establishing DNA methylation on the main promoter. Interestingly, these alternative promoters are also functional in the hypothalamus. It is currently unknown why these oocyte-specific alternative promoters are also functional in the hypothalamus. One scenario might be that some neuron cells within the hypothalamus might need similar functions as oocytes, establishing and/or maintaining DNA methylation on the main promoters. Although speculative at the moment, this should be one of important questions to be investigated in the near future.

## Materials and methods

### Ethics statement

All the experiments related to mice were performed in accordance with National Institutes of Health guidelines for care and use of animals, and also approved by the Louisiana State University Institutional Animal Care and Use Committee (IACUC), protocol #16–060.

### Mouse breeding

The C57BL/6J strain from the Jackson Lab (Stock No. 000664) was used in the current study. All the mice were housed at the DLAM (Division of Lab Animal Medicine) of LSU on a regular 12–12 dark-light cycle under a constant temperature 70°F and 50% humidity. All animals were given ad libitum access to water and Rodent Diet 5001. The nursing females were with Mouse Diet 5015. The mice were euthanized by CO2 asphixation in accordance with the rules and regulations set forth by the IACUC. Genomic DNA was isolated from either clipped ears or tail snips by incubating the tissues overnight at 55°C in the lysis buffer (0.1 M Tris-Cl, pH 8.8, 5 mM EDTA, pH 8.0, 0.2% SDS, 0.2 M NaCl, 20 μg/ml Proteinase K). The isolated DNA was subsequently used for genotyping. The sex of the pups was determined through PCR using the following primer set: mSry-F (5’-GTCCCGTGGTGAGAGGCACAAG-3’) and mSry-R (5’-GCAGCTCTACTCCAGTCTTGCC-3’).

### NGS-based 5’RACE experiments

Tissues were collected from hypothalamus, testis, liver, heart, and kidney from an adult male mouse (WT); ovaries were collected from an adult female (WT); a whole head was used from a one-day-old neonate (WT). The tissues were subject to total RNA isolation using the Trizol RNA isolation kit (Invitrogen). The resulting total RNA (2.5–5 μg) was mixed with gene-specific primers corresponding to the second exons for *Peg3*, *Gtl2*, *Dlk1*, *Igf2r*, *Snrpn*, *Zac1*, and *Myc* (S2 File), and reverse-transcribed using the M-MuLV reverse transcriptase (New England Biolabs, Cat. No. M0253S). The cDNA products were purified using phenol/chloroform extraction and ethanol precipitation. The 3′-ends of the purified cDNA was further modified by the tailing reaction using dGTP and terminal deoxynucleotidyl transferase according to the manufacturer’s protocol (New England Biolabs, Cat. No. M0315S). The tailed cDNA was amplified using two primers: the tail-long primer (5′ -GGTTGTGAGCTCTTCTAGATCCCCCCCCCCCCNN-3′) and internal gene-specific primers [22, 23]. The amplified cDNA was re-amplified with a set of nested primers: the tail-out primer (5′-GGTTGTGAGCTCTTCTAGA-3′) and additional internal gene-specific primers to increase the possibility to detect low abundant transcripts. The PCR products were further purified, multiplexed, and sequenced according to a next generation sequencing (NGS) protocol [22, 23]. The sequencing results from these NGS runs have been deposit to the SRA database (SRA Accession No. SRP156941).

### RT-PCR

Several sets of cDNA panel were generated using the total RNA isolated from various mouse tissues. In brief, the total RNA was isolated from each tissue using the Trizol RNA isolation kit (Invitrogen). The isolated total RNA (2.5–5 μg) was mixed with random hexamers, and subsequently reverse-transcribed using the M-MuLV reverse transcriptase (New England Biolabs, Cat. No. M0253S). The resulting cDNAs were used as templates for detecting the alternative and main transcripts for each imprinted gene. The information regarding the sequences of the primers used for these PCR are available (S2 File).

### Histone modification profiles and mammalian conservation plots

The histone modification profiles used for the current study was derived from the dataset of Release 3 (Aug 2012) of ENCODE/LICR, which contains a total of 130 ChIP-seq experiments on histone modifications. Mammalian conservation plots used for the current study was derived from the dataset of 30-Way Multiz Alignment and Conservation in the UCSC genome browser. This plot represents the results derived from comparison of genome sequences of 30 mammals, which was further refined with the phyloP program.

## Supporting information

S1 File Summary of the results from NGS-based 5'RACE experiments. This file contains the number of raw sequence reads for each gene, and also the summary of the mapping results showing the number of alternative transcripts in each library.
(XLSX)

S2 File Sequences and exon structures of the identified alternative transcripts. This file contains the information regarding the sequences and exon structures of the alternative exons of the imprinted genes, including *Igf2r*, *Mest*, *Zac1*, *Peg3*, *Snrpn*, and non-imprinted *Myc*. This file also contains the information regarding the sequences of all the primers used for RT-PCR analyses.
(DOCX)

S3 File Alternative promoter/exon of *Zac1*. This file contains the exon structure and histone modification profiles of the U1 alternative and E1 main promoters of *Zac1*. This file also contains the results from RT-PCR-based expression analyses of the U1 and E1 promoters.
(TIF)

S4 File Alternative promoter/exon of *Peg3*. This file contains the exon structure and histone modification profiles of the U1 alternative and E1 main promoters of *Peg3*. This file also contains the results from RT-PCR-based expression analyses of the U1 and E1 promoters.
(TIF)

S5 File Alternative promoters/exons of S*nrpn*. This file contains the exon structure and histone modification profiles of the U1, U2, U3 alternative and E1 main promoters of *Snrpn*. This file also contains the results from RT-PCR-based expression analyses of the U1, U2, U3, and E1 promoters.
(TIF)

S6 File Genomic duplication of the upstream region of mouse *Snrpn*. This file contains a set of dotplot results comparing the 450-kb genomic regions of *Snrpn* derived from mouse, rat, Chinese hamster, and human.
(TIF)
