# Supplementary material for: Enhancer-driven alternative promoters of imprinted genes
Source: PLoS One. 2018 Nov 30;13(11):e0208421. doi: 10.1371/journal.pone.0208421 (PMC6267961; doi:10.1371/journal.pone.0208421)
Supplement: S2 File — This file contains the information regarding the sequences and exon structures of the alternative exons of the imprinted genes, including Igf2r, Mest, Zac1, Peg3, Snrpn, and non-imprinted Myc. This file also contains the information regarding the sequences of all the primers used for RT-PCR analyses. (DOCX) [file pone.0208421.s002.docx]

**Alternative exons and RT-PCR primers for imprinted genes**

***Igf2r***

>Exon-U1 (chr17:12,984,397-12,984,550 in mm9)

GGCTAGCTCCGACACACTCTTTCCATGATGTCATCCACAATGGGAGAGAGGGAAAAGAATCTTTGAAAGAGCCAACATTATGCTGGAAGAAATTTGATAGTCAAACTTGGACGTAGTGGCTTGGGCCTGTAATCCCAGCACCCAGGAGGCTGAG

>Exon1

CGCTCCGGGGACGGCCACGGAGCGCCTCCTCGTCGCACTCCCCCCTGGCTCCAGTTCTCTCTCCTCTTTCTCCCTCCAGCTCCCGTTGCAGCTTCGACTCCGCTGTGGTGGCGCGACCGTGTCCCAGGCGCGGCTCCAAACGGCCAGCCGCCGTGAGCCCCACGCCACACGCGATGCGGGCCGTTCAGCTGGGACCGGTGCCCTCCGGGCCGCGCGTCGCGCTCCTGCCGCCGCTCCTGCTGCTGCTGCTCCTGGCGGCCGCGGGCTCCGCGCAGGCCCAGGCCGTCGACTTGGACGCCCTGTGCAG

>Exon2

CACTGAGCGGCAGTTCTCTGTCTTTAGGTCACACATACAGATAGCACTTGTAGGCCCACAGCTGGAAATACCCACATTTCCACAGACGTTGATCTTATAAACTGCATTGTTTTTGGAGTCAACAGCTTCCCATGTGTAA

Primers for RT-PCR

>Igf2r-RT-U1-F1

GGCTAGCTCCGACACACTCTTTCCA

>Igf2r-RT-U1-F2

TGATGTCATCCACAATGGGAGA

>Igf2r-RT-Ex1-F1

CTCTTTCTCCCTCCAGCTCCCG

>Igf2r-RT-Ex2-R3

GGAGTCAACAGCTTCCCATGTG

>Igf2r-RT-Ex2-R2

CAGCTGGAAATACCCACATTTC

>Igf2r-RT-Ex2-R1

GCGGCAGTTCTCTGTCTTTAGG

***Myc***

>Exon-U1 (chr15:61,810,640-61,810,833 in mm9)

ACAATAAACGAAGCCTTTGAAGCACTGTTAAACAAAAGAGCCATTTGTCTATGCTGGTGGCTACAAAGGAGAACAGGATTGCAGCAGAGGACTAAGAAAAGAAAGAGAAAATGCCAAACTGAGATGTGCCTGGACTTTACCGGGTCCTGCCCAGAGACCTTATATGCCAGGACCTAAGATCTAAAAGCCACTGG

>Exon1

CCCGCCCACCCGCCCTTTATATTCCGGGGGTCTGCGCGGCCGAGGACCCCTGGGCTGCGCTGCTCTCAGCTGCCGGGTCCGACTCGCCTCACTCAGCTCCCCTCCTGCCTCCTGAAGGGCAGGGCTTCGCCGACGCTTGGCGGGAAAAAGAAGGGAGGGGAGGGATCCTGAGTCGCAGTATAAAAGAAGCTTTTCGGGCGTTTTTTTCTGACTCGCTGTAGTAATTCCAGCGAGAGACAGAGGGAGTGAGCGGACGGTTGGAAGAGCCGTGTGTGCAGAGCCGCGCTCCGGGGCGACCTAAGAAGGCAGCTCTGGAGTGAGAGGGGCTTTGCCTCCGAGCCTGCCGCCCACTCTCCCCAACCCTGCGACTGACCCAACATCAGCGGCCGCAACCCTCGCCGCCGCTGGGAAACTTTGCCCATTGCAGCGGGCAGACACTTCTCACTGGAACTTACAATCTGCGAGCCAGGACAGGACTCCCCAGGCTCCGGGGAGGGAATTTTTGTCTATTTGGGGACAGTGTTCTCTGCCTCTGCCCGCGATCAGCTCTCCTGAAAAGAGCTCCTCGAGCTGTTTGAAGGCTGGATTTCCTTTGGGCGTTGGAAACCC

>Exon2

CAGACAGCCACGACGATGCCCCTCAACGTGAACTTCACCAACAGGAACTATGACCTCGACTACGACTCCGTACAGCCCTATTTCATCTGCGACGAGGAAGAGAATTTCTATCACCAGCAACAGCAGAGCGAGCTGCAGCCGCCCGCGCCCAGTGAGGATATCTGGAAGAAATTCGAGCTGCTTCCCACCCCGCCCCTGTCCCCGAGCCGCCGCTCCGGGCTCTGCTCTCCATCCTATGTTGCGGTCGCTACGTCCTTCTCCCCAAGGGAAGACGATGACGGCGGCGGTGGCAACTTCTCCACCGCCGATCAGCTGGAGATGATGACCGAGTTACTTGGAGGAGACATGGTGAACCAGAGCTTCATCTGCGATCCTGACGACGAGACCTTCATCAAGAACATCATCATCCAGGACTGTATGTGGAGCGGTTTCTCAGCCGCTGCCAAGCTGGTCTCGGAGAAGCTGGCCTCCTACCAGGCTGCGCGCAAAGACAGCACCAGCCTGAGCCCCGCCCGCGGGCACAGCGTCTGCTCCACCTCCAGCCTGTACCTGCAGGACCTCACCGCCGCCGCGTCCGAGTGCATTGACCCCTCAGTGGTCTTTCCCTACCCGCTCAACGACAGCAGCTCGCCCAAATCCTGTACCTCGTCCGATTCCACGGCCTTCTCTCCTTCCTCGGACTCGCTGCTGTCCTCCGAGTCCTCCCCACGGGCCAGCCCTGAGCCCCTAGTGCTGCATGAGGAGACACCGCCCACCACCAGCAGCGACTCT

Primers for RT-PCR

>Myc-RT-U1-F1

ATGCTGGTGGCTACAAAGGAG

>Myc-RT-U1-F2

GGAGAACAGGATTGCAGCAGA

>Myc-RT-Ex1-F1

CGCGATCAGCTCTCCTGAAAAG

>Myc-RT-Ex2-R3

GTTGGTGAAGTTCACGTTGAG

>Myc-RT-Ex2-R2

CGCTCTGCTGTTGCTGGTGATAG

>Myc-RT-Ex2-R1

CTTCCAGATATCCTCACTGGGC

***Mest***

>Exon-U2 (chr6:30,673,546-30,673,646 in mm9)

CATACTTTTCAGGCTACCCAGGAGCGCGGCTGCTCCCAGGGCTGCATGCGCAGCCTCCAAGCCTTCATGCATGGGCATTGGCTCTCCCAACCCAGCCACAG

>Exon-U1 (chr6:30,683,506-30,683,613 in mm9)

GGGGTAGAGAGAAAAAGTGTGGAAGGCTGCGGTCTAGTCTTCCTTCTTGGGCAGCTGGGAAGAGAAAGCCAGCTTGTTTGGAAGTCGCTGTTCCTTAGAGGGCCTGTG

>Exon1

CCAGCACATCCCGGTGCTTCTTCTCAGGCGCAGCAGCTTTCCTCTGCGGCAGCCGCACCTCGCCAAACGGCGTAGTGCTGCAGGCTCGCCCGAGTTGCTGCTTGCTGCCTCTGCTGCCGCTGCCGCGGGCCGCCCTGCGCGGACCGTAGGCTGCGCAGACGCCACCTCCGATCCTGTATCGCTGCGGGCGCCTCGGCGCGCCCTGTGATCCGCAATCCTGCGGCGGGCGGCATGGGATAATGCGGCCATGGTGCGCCGAGATCGCTTGCGCAG

>Exon2

GATGAGAGAGTGGTGGGTCCAAGTAGGGCTCCTGGCTGTGCCCTTGCTGGCTGCGTACCTGCACATCCCGCCCCCTCAGCTCTCCCCTGCTCTGCACTCATGGAAGACTTCTGGCAAGTTTTTCACCTACAAAGGCCTACGCATCTTCTACCAAG

Primers for RT-PCR

>Mest-RT-U1-F1

GGGGTAGAGAGAAAAAGTGTGGA

>Mest-RT-U2-F1

CATACTTTTCAGGCTACCCAGGA

>Mest-RT-E1-F1

GCCCTGTGATCCGCAATCCT

>Mest-RT-E2-R1

CTTGCCAGAAGTCTTCCATGA

***Snrpn***

>U3-450kb (chr7:67,594,964-67,595,063 in mm9)

AGTCAAGTCAGTGCAGCAGGTCCTGCTCAGAGAAGAGGCAGCCAAAGATGCCTGTCACATCCACCCTAGGAAAGGACAGGAAGGCAATACAGCAAACAAG

>U2-130kb (chr7:67,285,027-67,285,105 in mm9)

CCTGCTCAGAGAAGAGGCAGGCAAACATGCCTCTCACATCCACCCTAGGAATGGTCAGCAAGGCAATGCAGCAACCAAG

>U1-90kb (chr7:67,242,416-67,242,495 in mm9)

AGTGCAGCAGGTCCTGCTGAGCCAAAGATGCCTGTCACATCCACCCTAGGAATGGTCAGCAAGGCAATGCAGCAACCAAG

>Exon1

GGCAAAAATGTGCGCATGTGCAGCCATTGCCTGGGACGCATGCGTAGGGAGCCGCGCGACAAACCTGAGCCATTGCGGCAAGACTAGCGCAGAGAGGAGAGGGAGCCGGAGATGCCAGACGCTTGGTTCTGAGGAGTGATTTGCAACGCAATGGAGCGAGGAAG

>Exon2

GGATCGCTTACACTTGAGAAGAACTACTGAACAGCACGTGCCCGAGGTCGAGGTCCAGGTCAAACGTCGAAGGACAGCCTCACTGAGCAACCAAGA

Primers for RT-PCR

>U3-450-F1

CCTGCTCAGAGAAGAGGCAGCCA

>U3-450-R1

CCTGCTAGTGCATGCTGCTTG

>U2-130-F1

CCTGCTCAGAGAAGAGGCAGGCA

>U1-90-F1

GTGCAGCAGGTCCTGCTGAGCCA

>Snrpn-RT-EX1F1

AAACCTGAGCCATTGCGGCAAGAC

>Snrpn-RT-EX2R1

CTTCGACGTTTGACCTGGACCT

>Snrpn-RT-EX2R2

GTAGTTCTTCTCAAGTGTAAGC

***Zac1***

>Exon-U1 (chr10:12,780,311-12,780,665 in mm9)

CAGAAAACAGACCACCTGGCGAGTGTGCCAGGCATTTTAGCAAGGCTTCTCACAGGCTTAATTCTTGCAACCAGTTTAAATGAAGACTAGGGCAGGGTGTTGGTGCCTGAAGCTAATGACTATTGAGGGCTGGGCTTCCTTATTCGGAAGCCTTGGACAGCCCAGAACCTTAAATTTGTCTGGAAGATTTTCTTCCCAGCTTTTAAGCTCCAGATCCCAGAGCACATGGATCCGCTCATGGGCAGGTATCACGACCTCCCCACTTCCGCAGAAAGCGGTAAAGTTAAGCGGCCAAAGGTTGTACACAAATAAGGACCATGGAAGTATCTGCACCCAGATGGCCTAACCCCTAAAG

>Exon1

GGACCGCCCCGAGCCTTGATTTAGCCGGGGCTGGGGCGTTCTCCAACCTCACTCGCCTGGCAGGCGGGAGAACGCTCGGGGAGTTGCGGCCGCGGGCACCGGGCTCGCGGCTATCGGGACTGGAGAGCAAGCGGGCATCTCCTGGGCGCCGTCATGGCTGCTTAGGCTGCGCCTGCCTGCGGATCGCGGATCCGGGATCGGAGATCTGACGGCGACGCCTGAGTCCGGCTAGGGTAG

>Exon1.a

GTTGTTCCTGCTTGATTGCTTCAGCGTGCCATCGGCTTC

>Exon2

GTATTTGCATAGGAGTCAGAGGAGTTAATCTT

>Exon3

CTCTTCTCACAGGTTTGAGTCTTCAGACTTCTACAGAACTCCATAATATCTGCCTCACAGCTGGCTTTCCTGCTCTCACAGAAG

Primers for RT-PCR

>Zac1-RT-3

AGCGGCCAAAGGTTGTACACA

>Zac1-RT-4

CCTCTGACTCCTATGCAAATAC

>Zac1-RT-2

GGTCTGGAGGTGGTTCTTCA

>Zac1-RT-1

TTCGTCACCCTGGAGAAGTT

***Peg3***

>Exon-U1 (chr7:6,703,770-6,703,946 in mm9)

GCAGGGAAGCCTCCATCCGTTTGTCTTTAGGCTTTCCATTCCCAGCATCT

CATAGAAGTGTAATCTTCGCTATTTACCATCCCTTCTTTTAACTAATCAA

GGTTGCAGTTTTATCAGGAGTTTGGTATCCCTCTTAAATGGTTTGAGAAG

CTCAGAACTGCACTGCAGGGGATTCAG

>Exon-U0

ATACAATGATACAACATGGCCAGCCTTAACCACAGATATAATGATGCAAC

ATGACCAGCAGTTCCTCCCACTCCAATTGAAGCCCTGAGCTCCTGCACAC

TCCAGCTTCACTGCCATGATGGACCCTACCCTGGAACT

>Exon1

AGACGCTGGGGAGTCAGGAGTCGCGGGAGGACGAGCATCGGAGGAGAAGC

GGAGAGATGTCCACCCTGGGCTGGTGGCGCCGCCGGGCGCCCGGTTCAGT

GTGGGTGCACTAGACTGCCGACCCTGGTCGGGGTGTGTGCGTAGAGTGCT

GTGCTCCGGGAG

>Exon2

CCCTACCTTCTTGATCTTCTATCCTTTTTGGAGACAACTGGCAAGAGGAA

GACTAGGTCCTCCAACAGCTAGGTGGCTGGTCCAG

>Exon3

GCAGGCCTTCCCAGCAAGGGGAGATCAGTTGATCATCCCTGAAACGCTCA

AGCCCTTGGGTGTGAGCAAAACAGACAACTGTGAAAAACTCACCACTCCG

TTGGAGAGTTTCAAGATGTACCATCACGAAG

Primers for RT-PCR

>Peg3-RT-U1-F

AGGGAAGCCTCCATCCGTTTGTC

>Peg3-RT-1a

GGTTCAGTGTGGGTGCACTAGACT

>Peg3-RT-1b

GCTCACACCCAAGGGCTTGAGCG

***Gtl2***

>Exon1

AGCACAGAAGACGAAGAGCTGGAATAGAGCTCGCCTCGGCTCTGCTGGCCTTGGCTGCAGCTCTTCCAGAAACCCGGGGCGCCCACAGAAGAATCTCTTACCTG

>Exon2

GCTCTCTCTTCAGGGATGACATCATCGGCTCACACCAGTCTTCCAGGACCACCTTCTGGATGCCAAGGCTGCTGCTCGAGTACCTGCTGTGCACCTCTACCTCCTGAGCCAAGGAGCACGGATTCCAGGAACCCACTACCATACAGAGCAACTCCTTGTGGACCCCCCTGGGATCAGGACAGCGAGGGACAAGCGACAAAGAGGATCATCAGTGGCCAGCTAGTTTCTCTGGGGTTCAAACCTTGAACCAGTGCCCTAGTGAGGGGGCACTGGCCATGGCCCTTGACCTTTGCTCTGCTTGTGTCTTGAGTCTGAGCCCTTTCCTGTACATCTGTGCTCGTGTTCATCTGCTAGTGAACTGGAGTGCTGCCCTCCCCGAGGAGGGTCGTCCCTTGTGACTGATCATGCTGTCCTAACAATGTCCTGAGCAAAAGGGTCCCTTTGGGAACCTCTCAGGAGGGGGACCCGGGTCAGGGGCGACCAGCATCTTGCTGGCAACTCCGTGGGTGGGGTGGGGTGGGGTGCTTCCTTCTGGAATGAGCACGTGGCTGACCCCCCAAGGCATGTCCCCTCCCCCTCCTCCACCCACCTTCTCGGAGATGTCCCTTTTGGGGTAGTGGGGACATTAGGAGCAACCTCCTAGGGTTGTTGTGAGAATTAAATGAACTGCAGCAGCCTGAGGCAGGGCTGGGCAGAGACCTCAGCACATGTTTGTTGAAAGGTTTGCAGGTGGATCTAGTCCTCCCGTTCATGGCTCATGTGTCTCAACCATTCTCTCGCAGACTCCTGCAGCCCCTATGCCCAGGGCTCTCCTTGCGCCAG

>Exon3

AGGTAGGTGGGAAAGAGAACTGGGAGAGCCCGGACTCACTCATGAGATTGAACTTAAATTCACACGGAGGACACTTGGACTCTTGCCACATTAG

Primers for RT-PCR

>Gtl2-RT-Ex1-F1

CGCCCACAGAAGAATCTCTTAC

>Gtl2-RT-Ex2-R3

ATGTCATCCCTGAAGAGAGAGC

>Gtl2-RT-Ex2-R2

ACTGGTGTGAGCCGATGATGTC

>Gtl2-RT-Ex2-R1

TAGAGGTGCACAGCAGGTACTC

***Dlk1***

>Exon1

GACAGTACGAAAAGGCGGCGCGCGGGCCGCAGCGGCAGCTCCTCGGCAGCCGCACTTAGCAGCGTGCGCCCCGGTGCAACCCTGGCTTTCTTCCCGCTGGACGCCCGTGCCCCCTTCGTGGTCCGCAACCAGAAGCCCAGCGCAGCCCCCGGAGCAGCCCCTGCACCGCCTCCGCTCCCCGGACCGCGACCCAGGCCGCCCCGAGATGATCGCGACCGGAGCCCTCCTGCGCGTCCTCTTGCTCCTGCTGGCTTTCGGCCACAGCACCTATG

>Exon2

GGGCTGAATGCGACCCACCCTGTGACCCCCAGTATGGATTCTGCGAGGC TGACAATGTCTGCAG

Primers for RT-PCR

>Dlk1-RT-Ex1-F1

CTTTCGGCCACAGCACCTATG

>Dlk1-RT-Ex2-R3

TCACAGGGTGGGTCGCATTCAG

>Dlk1-RT-Ex2-R2

CAGAATCCATACTGGGGGTCAC

>Dlk1-RT-Ex2-R1

CTGCAGACATTGTCAGCCTCGC

***Grb10***

>Exon1-M (chr11:11,937,195-11,937,424 in mm9)

CATCGAGGGGGTGGGGTGCGGGGAGGCGGCAGGAAGGGAAGGGCGCTGCGACCAGTGGCGGGCGGGATTCGCGTTCCGAGACCCACGGGAGCACGAAGTTTCCGCGCACCGTCTCACGCACGGCGACTGGGACCGTCCAGTGTCCGGCTTTGCCTTCGGTTTTTCTCCGTTGTGACTCGTGCAACGTGTGGCCAGCGGCCACGCGGAGGCGACGAGGAGCTGCACGTCAG

>Exon1-P (chr11:11,925,841-11,926,327 in mm9)

GGTTTGTAGGCGCGTGCCAGCGCGTGCAGGCCTGTGTTGGCGCGTGCCGGCTTATGTTGGCGCGTGCCGGCCTGCCGCGATCATTCGTCTCTGAGCGGCACGGCCCTGTCGGTTCGTTTAGGAGCTAGTTCGCTGTGCCGGTTTGTGTTGGCGCCTGTGGGCCAGTGTTGGCGCATGTAACACGTATGTTGGCGCGTGTTGCCGCGTGTTGGCCCGTGTTGCTGCGTGTCAGCGCATGTTGCCGCATGTCGGTGCGTGCCTGCTGGGCCTCCGCGCTCCTCTAGGCTCCAGAGCCCTTTTTCTGAGCCGTAGGCCCTTAGCTGGGGCGGAGCCTTGGCGAACCCCAGCGAGCCGCGGGGCCGAGCGCTGTCCATGAGGAGCGCTGTCCACGCGCTGGGTGCTGCCGGGTTCGGGAGCTGTCCACTGGCCCCGGTAGCGCGCACGCAGCGACCCCGTGTGGCGAAGTCTGTGCTAGCCGGCTCTCCAG

>Exon2

GGACAAAGTGGGGCAGTCAACGTCCAAACCCGAAAACCTAGCTAAGTCTGGGTTTTCGCCACAACAAAGAAGCCAACCAG

Primers for RT-PCR

>Grb10-RT-3

GAGCACGAAGTTTCCGCGCA

>Grb10-RT-4

CTGGTTGGCTTCTTTGTTGTGG

>Grb10-RT-5

CGGGTTCGGGAGCTGTCCACT

>Grb10-RT-6

CTGGTTGGCTTCTTTGTTGTGG
