# Supplementary figures and images for: Enhancer-driven alternative promoters of imprinted genes
Source: PLoS One. 2018 Nov 30;13(11):e0208421. doi: 10.1371/journal.pone.0208421 (PMC6267961; doi:10.1371/journal.pone.0208421)

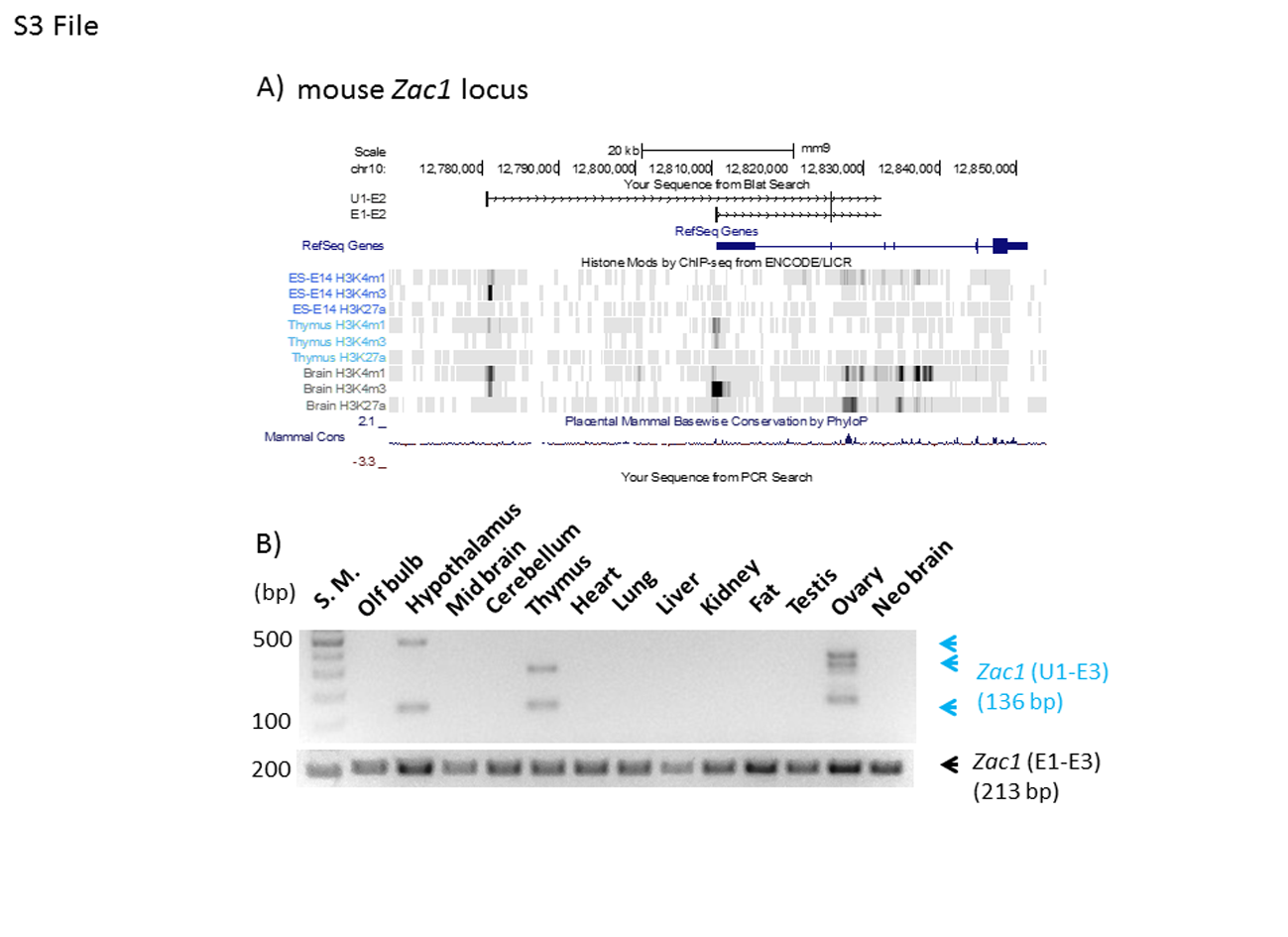

Supplement: S3 File — This file contains the exon structure and histone modification profiles of the U1 alternative and E1 main promoters of Zac1. This file also contains the results from RT-PCR-based expression analyses of the U1 and E1 promoters. (TIF) [file pone.0208421.s003.TIF]

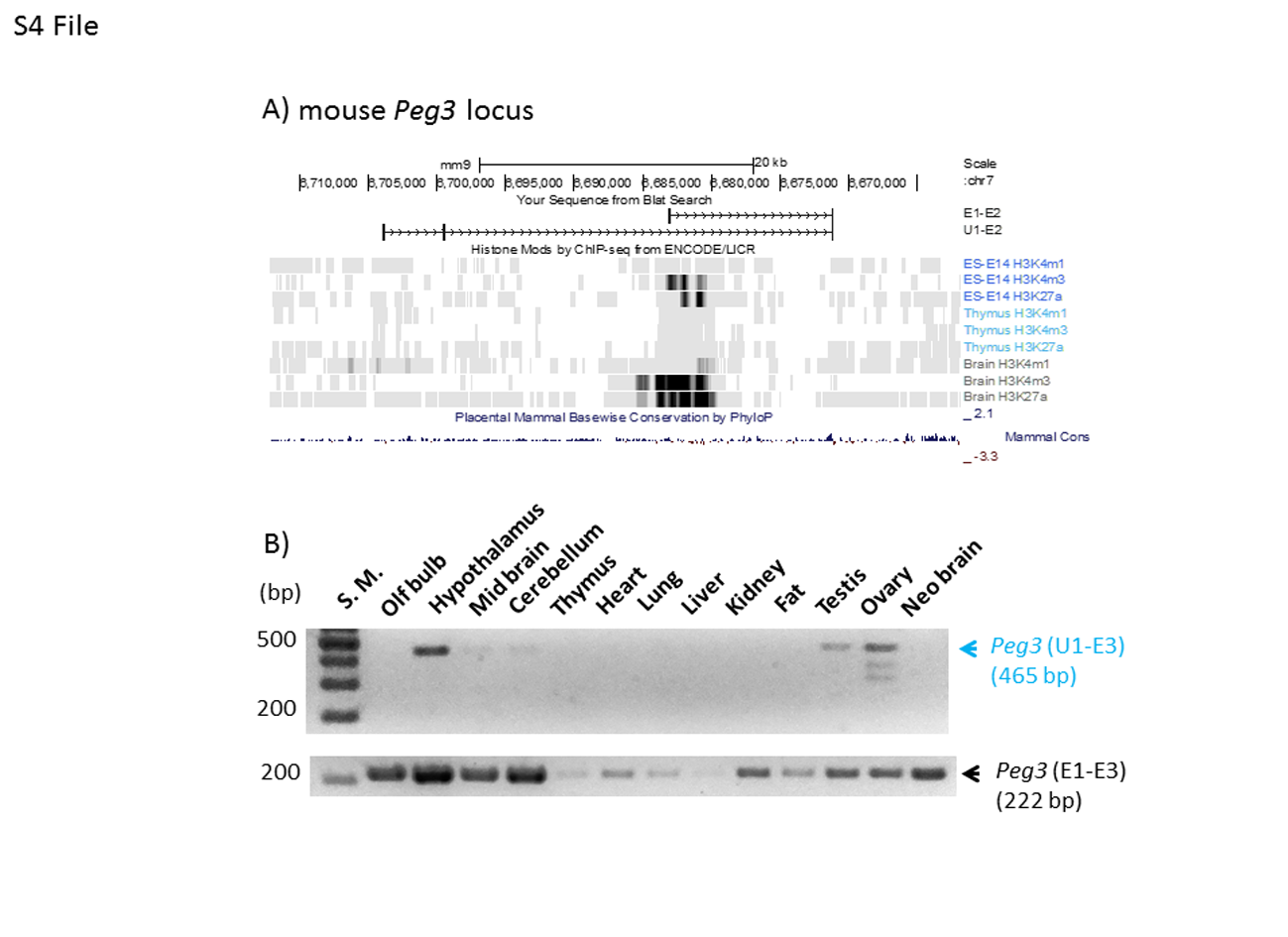

Supplement: S4 File — This file contains the exon structure and histone modification profiles of the U1 alternative and E1 main promoters of Peg3. This file also contains the results from RT-PCR-based expression analyses of the U1 and E1 promoters. (TIF) [file pone.0208421.s004.TIF]

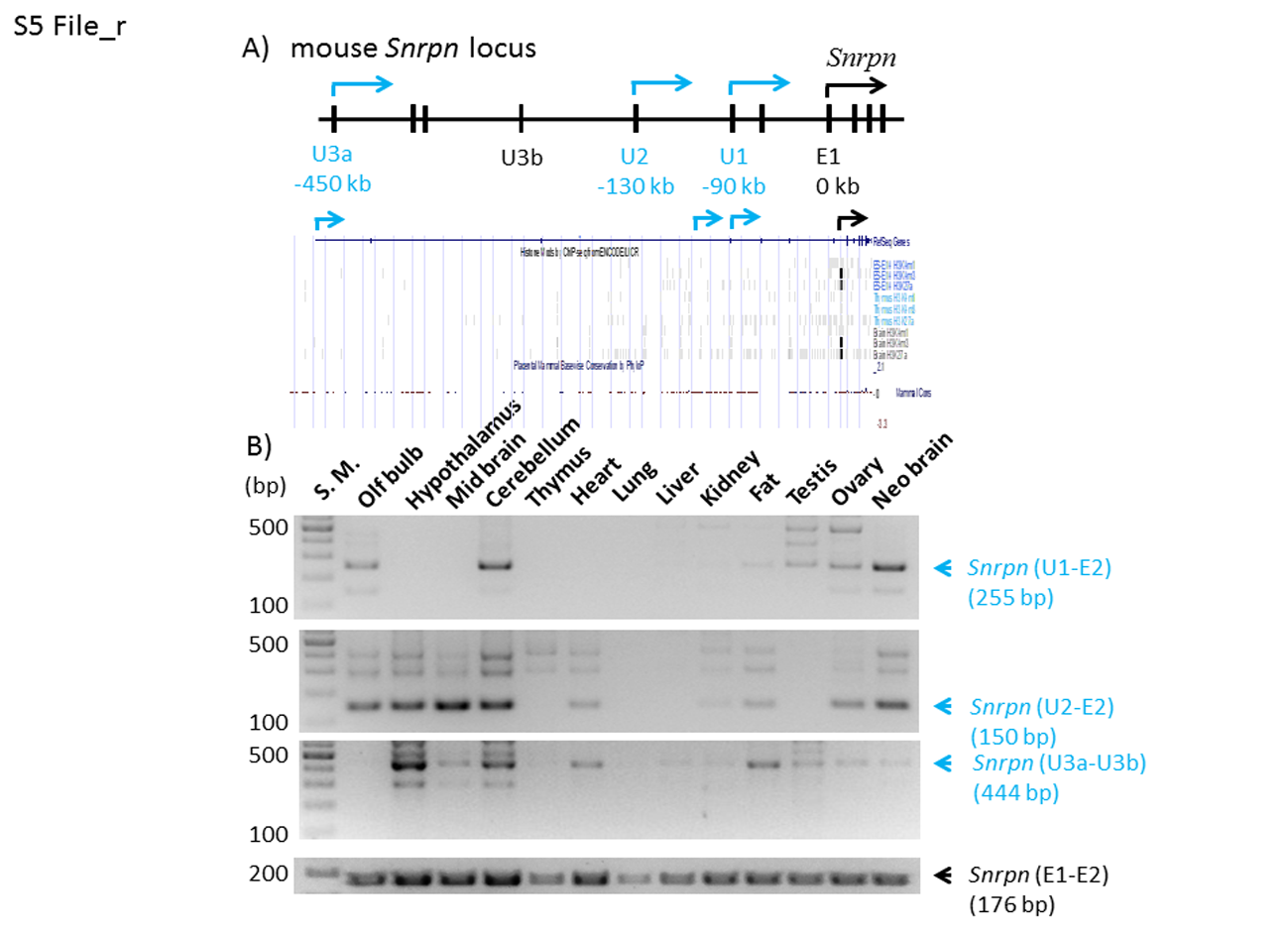

Supplement: S5 File — This file contains the exon structure and histone modification profiles of the U1, U2, U3 alternative and E1 main promoters of Snrpn. This file also contains the results from RT-PCR-based expression analyses of the U1, U2, U3, and E1 promoters. (TIF) [file pone.0208421.s005.tif]

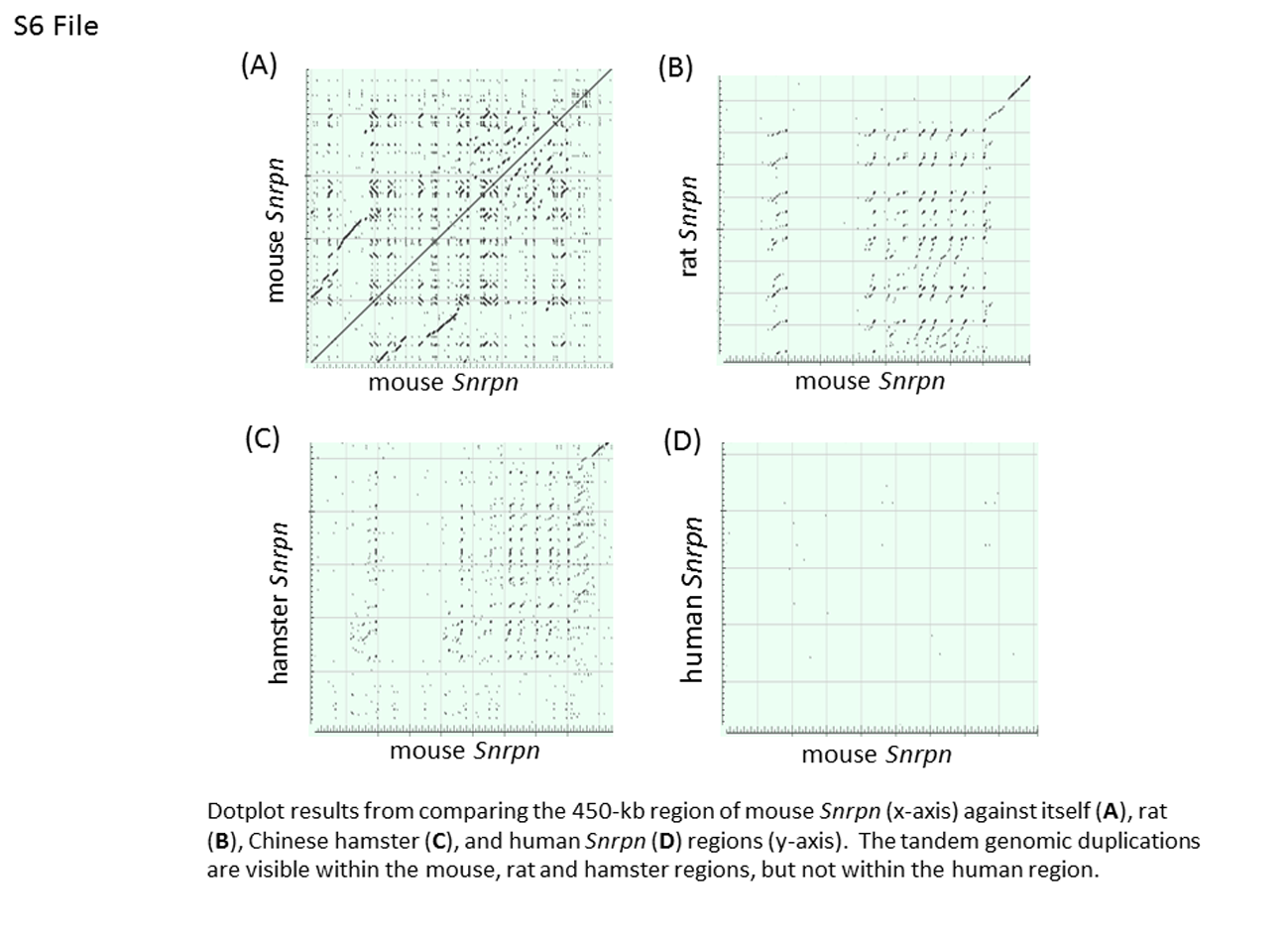

Supplement: S6 File — This file contains a set of dotplot results comparing the 450-kb genomic regions of Snrpn derived from mouse, rat, Chinese hamster, and human. (TIF) [file pone.0208421.s006.TIF]
